# Mild Inactivation of RE-1 Silencing Transcription Factor (REST) Reduces Susceptibility to Kainic Acid-Induced Seizures

**DOI:** 10.3389/fncel.2019.00580

**Published:** 2020-01-10

**Authors:** Emanuele Carminati, Federica Buffolo, Anna Rocchi, Caterina Michetti, Fabrizia Cesca, Fabio Benfenati

**Affiliations:** ^1^Center for Synaptic Neuroscience and Technology, Istituto Italiano di Tecnologia, Genova, Italy; ^2^IRCCS Ospedale Policlinico San Martino, Genova, Italy; ^3^Department of Life Sciences, University of Trieste, Trieste, Italy

**Keywords:** RE-1 silencing restriction factor (REST), epilepsy, gene transcription, light-oxygen-voltage (LOV) domain, paired-amphipathic helix 1 (PAH1) domain, kainic acid

## Abstract

RE-1 Silencing Transcription factor (REST) controls several steps in neural development by modulating the expression of a wide range of neural genes. Alterations in REST expression have been associated with the onset of epilepsy; however, whether such alterations are deleterious or represent a protective homeostatic response remains elusive. To study the impact of REST modulation on seizure propensity, we developed a tool for its negative modulation *in vivo*. The tool is composed of the paired-amphipathic helix 1 (PAH1) domain, a competitive inhibitor of REST activation by mSin3, fused to the light-oxygen-voltage sensing 2 (LOV2) domain of *Avena sativa* phototropin 1, a molecular switch to alternatively hide or expose the PAH1 inhibitor. We employed the C450A and I539E light-independent AsLOV2 variants to mimic the closed (inactive) and open (active) states of LOV2-PAH1, respectively. Recombinant AAV1/2 viral particles (rAAVs) allowed LOV2-PAH1 expression in HEK293T cells and primary neurons, and efficiently transduced hippocampal neurons *in vivo*. mRNA expression analysis revealed an increased expression of several neuronal genes in the hippocampi of mice expressing the open probe. AAV-transduced mice received a single dose of kainic acid (KA), a treatment known to induce a transient increase of REST levels in the hippocampus. Remarkably, mice expressing the active variant displayed a reduced number of KA-induced seizures, which were less severe compared to mice carrying the inactive probe. These data support the validity of our tool to modulate REST activity *in vivo* and the potential impact of REST modulation on epileptogenesis.

## Introduction

The specification of cell identity during central nervous system development is regulated by positive and negative transcriptional regulators that act simultaneously to shape the cell-specific transcriptome. The RE1-silencing transcription factor (REST), also known as neuron-restrictive silencer factor (NRSF), is a transcriptional repressor that binds a specific 21 bp consensus sequence named repressor element 1 (RE-1; Chong et al., 1995; Schoenherr and Anderson, 1995). REST is a member of the Kruppel-type zinc finger transcription factor family, whose repressive functions are mediated by two repressor domains: the N-terminal domain interacts with Sin3 (Grimes et al., 2000), while the C-terminus recruits CoREST (Andrés et al., 1999; Ballas et al., 2001). In turn, each co-repressor recruits other associated proteins and chromatin remodeling factors, including histone deacetylases (e.g., HDAC1/2), demethylases (e.g., LSD1), and methyltransferases (e.g., G9a) that mediate the transcriptional repression of target genes (Ballas et al., 2005). Genome-wide sequencing analyses identified several thousands putative RE-1 sites (Mortazavi et al., 2006; Johnson et al., 2007; Jothi et al., 2008), most of which are found in neuron-specific genes (Bruce et al., 2004; Johnson et al., 2007; Otto et al., 2007). Indeed, REST represses the expression of various channels, such as sodium (Chong et al., 1995; Pozzi et al., 2013), calcium (Ariano et al., 2010; van Loo et al., 2012) and potassium channels (Cheong et al., 2005). Moreover, it mediates the transcriptional downregulation of the KCC2 chloride transporter, which is involved in the GABAergic switch from excitatory to inhibitory transmission during neuronal maturation (Yeo et al., 2009). Likewise, REST has been shown to downregulate the expression of Grin2b and GluR2, which code for the NMDA and AMPA receptor subunits, respectively (Calderone et al., 2003; Qiang et al., 2005; Rodenas-Ruano et al., 2012), further supporting its fundamental role in the modulation of genes involved in synaptic activity and plasticity. REST is also involved in the control of neurotransmitter release, whereby it represses several genes involved in neurosecretion, like SNAREs (D’Alessandro et al., 2009), and in synaptic vesicle trafficking, like synapsin 1 (Paonessa et al., 2013).

Because of its pleiotropic functions, alterations of REST expression and/or activity have been described in a wide spectrum of disorders, including Alzheimer’s (Lu et al., 2014) and Huntington’s disease (Zuccato et al., 2003, 2007) and various types of cancer, where it can act as either tumor suppressor or oncogene, depending on the cellular context (Negrini et al., 2013). In the brain, increased REST levels have been observed after epileptic or ischemic insults (Baldelli and Meldolesi, 2015). In epilepsy, the role of REST is still debated. On the one hand, it seems to have a protecting role as it maintains cell homeostasis by downregulating genes like BDNF (Timmusk et al., 1999; Garriga-Canut et al., 2006); on the other hand, it appears to participate in the induction of the disease, mediating epileptogenesis by inhibiting genes such as HCN1, a hyperpolarization-activated, cyclic nucleotide-gated channel, involved in synaptic transmission and neuronal excitability (McClelland et al., 2011, 2014; Patterson et al., 2017). *In vitro* and *in vivo* studies with kainate, an agonist of glutamatergic receptors, have shown the upregulation of REST levels in hippocampal and cortical neurons (Palm et al., 1998; Hu et al., 2011; McClelland et al., 2014), but whether such increase is protective or deleterious is still not understood. In a rat model of global ischemia, REST is strongly upregulated in post-ischemic CA1 neurons, and linked to neuronal death through the suppression of the AMPA receptor subunit GluR2 (Calderone et al., 2003), modulation of calcium permeability and silencing of the μ-opioid receptor 1 (MOR-1; Formisano et al., 2007). The role of REST in the onset and development of epileptogenesis was addressed by inducing the conditional deletion of REST in mice. The progression of kindling-induced seizures was faster in mice bearing the Calcium/calmodulin-dependent protein kinase II (CaMKII)-Cre driven REST deletion, with a concomitant worsening in mossy fiber sprouting (Hu et al., 2011). In contrast, animals bearing the neuron-specific enolase (NSE)-Cre driven REST deletion were characterized by attenuated susceptibility to pentylenetetrazol (PTZ)-induced seizures (Liu et al., 2012). More recently, the transient block of REST activity *via* a decoy strategy enabled the rescue of the memory impairment induced by febrile seizures (Patterson et al., 2017). These conflicting data could be explained by the different seizure models and/or by the different cell populations where REST was deleted. This suggests that REST may have different functions in the signaling pathways activated by the various convulsants, and/or in the various targeted cell types.

In this work, we have addressed the role of REST in the modulation of kainic acid (KA)-induced seizures. To do so, we have exploited a molecular tool composed of the paired-amphipathic helix 1 (PAH1) domain, a competitive inhibitor of REST activation by mSin3, fused to the light-oxygen-voltage sensing 2 (LOV2) domain of *Avena sativa* phototropin 1, a molecular switchable to alternatively hide or expose the PAH1 inhibitor (Paonessa et al., 2016). Our previous work demonstrated that the LOV-PAH1 probe efficiently controls the expression of REST target genes in primary neuronal cultures, thus modulating network excitability *in vitro* (Paonessa et al., 2016). Here, we performed intra-hippocampal injection of AAVs expressing LOV2-PAH1 and showed that a mild and long-term inhibition of REST activity reduces the susceptibility of mice to develop KA-induced seizures *in vivo*. Overall, our data suggest that REST represents a potential target for therapeutic approaches addressed to pathologies characterized by network hyperexcitability, such as epilepsy.

## Materials and Methods

### Materials

All biochemical reagents and drugs were from Sigma-Aldrich unless otherwise specified. Tissue culture reagents and media were from Gibco-Invitrogen (Thermo-Fisher Scientific, Waltham, MA, USA) or Sigma-Aldrich (Milano, Italy).

### Animals

All animals used in this study were mice on the C57BL/6 background (Charles River, Calco, Italy). All experiments were carried out in accordance with the guidelines established by the European Community Council (Directive 2010/63/EU of 22 September 2010) and were approved by the Italian Ministry of Health (Authorization #73-2014-PR on Dec 5, 2014). Hippocampal stereotaxic injections (from Bregma: AP 2.2, LAT 1.5; from brain: Z 1.65) were performed on C57Bl6/J (12–24 weeks) male mice and neurons transduced with either the “closed state” inactive AsLOV2 (C450A)-PAH1 or the “open state” active AsLOV2 (I539E)-PAH1 variant. Anesthesia was induced by brief exposure to 4% isoflurane and maintained by intraperitoneal (IP) injection of the following anesthetic mixture: ketamine 100 mg/kg, medetomidine 0.65 mg/kg, acepromazine 1.5 mg/kg, atropine 0.05 mg/kg. Mice were placed in a stereotaxic frame and the head adjusted to a flat-skull position. A small craniotomy was performed bilaterally at the injection coordinates indicated above and rAAV1/2 particles carrying AsLOV2 (C450A)-PAH1 or AsLOV2 (I539E)-PAH1 were injected in the hippocampus *via* a glass pipette (0.65 μl–0.75 μl/site at a flow rate of 0.1 μl/min). The injection pipette was left in place for at least 5 min at the end of each injection to allow the complete diffusion of the virus. After injection, mice were returned to their home cage and administered with atipamezole (0.65 mg/kg, IP) to speed up recovery from anesthesia. Mice were allowed to recover for at least 4 weeks before behavioral experiments.

### Cloning and AAV Production

To obtain pAAV-CMV_AsLOV-His_Ires GFP, 20 ng of pcDNA3.1_AsLOV2_His (Paonessa et al., 2016) were PCR-amplified using Pfu DNA polymerase (© BiotechRabbit, Hennigsdorf Germany), using primers #1 and #2 (see below). PCR conditions were: 95°C, 5 min; (95°C, 30 s; 60°C, 30 s; 72°C, 1 min) for 27 cycles; 72°C, 5 min and 4°C, ∞. PCR products were digested using Bam HI and Sal I enzymes (NEB, Ipswich, MA, USA), cloned directly in pAAV-IRES-hrGFP Vector (Agilent, Santa Clara, CA, USA), digested with the same enzymes and transformed into TOPTEN cells. Positive colonies were verified by DNA sequencing. To obtain pAAV-CMV_AsLOV-PAH-His_Ires GFP, we proceeded as described above, but starting from pcDNA3.1_AsLOV2_PAH1b_His (Paonessa et al., 2016).

Primer #1 (Fw)  5′CCACCATGGGCGAATTCTTG3′

Primer #2 (Rv)  5′ATCCGTCGACTCACTTCAATGGTGATGGTGATGATGAC3′

AAV1/2 expressing pAAV-CMV_AsLOV-His_Ires GFP and pAAV-CMV_AsLOV-PAH-His_Ires GFP were generated as previously described (McClure et al., 2011). Briefly, human embryonic kidney (HEK)293T cells were co-transfected with the required AAV vector together with the plasmids pRV1, pH21 and pHelper using a Ca^2+^ phosphate method. Forty-eight hours post-transfection, cells were harvested and lysed, and viruses purified over heparin columns (GE HealthCare Life Science, Milano, Italy). Viral vectors were titrated at concentrations ranging from 1 × 10^11^ to 1 × 10^12^ transducing units (TU)/ml and used at a multiplicity of infection (MOI) of 10,000. The efficiency of infection, estimated by counting neurons expressing GFP protein with respect to the total number of cells stained with DAPI, ranged between 70% and 90%.

### Cell Culture and Transfection/Infection

#### Immortalized Cells

HEK293T cells were cultured in DMEM (#11965-092) supplemented with 10% (vol/vol) fetal bovine serum (FBS), glutamine (2 mM), and antibiotics, in a humidified 5% CO_2_ atmosphere at 37°C. For immunostaining experiments, 180,000 cells were seeded on 24-mm coverslips and the day after were transiently transfected with Lipofectamine 2000 (Life Technologies) following standard transfection procedures.

#### Primary Neurons

Primary cortical neurons were obtained from E18 embryos derived from crosses of C57BL/6 wild type mice. Mice were mated overnight and separated the following morning. The development of the embryos was timed from the detection of a vaginal plug, which was considered day 0.5. Cortices were dissected in ice-cold phosphate-buffered saline (PBS), incubated with trypsin (0.125%) for 15 min at 37°C, and mechanically dissociated. Neurons were plated in Neurobasal medium containing 10% horse serum, 2 mM glutamine and antibiotics (plating medium). After 3 h, the medium was removed and replaced with Neurobasal containing 2% B27 supplement, 2 mM glutamine and antibiotics (maintenance medium). Neurons were infected at 5 DIV. Experiments were performed 12 days after infection (17 DIV).

### Western Blotting

Both tissues and cells were lysed in RIPA buffer (10 mM Tris-HCl pH 7.4, 140 mM NaCl, 1 mM EDTA, 0.5 mM EGTA, 1% Triton X-100, 0.1% SDS, 0.1% sodium deoxycholate) supplemented with proteases and phosphatase inhibitors [complete EDTA-free protease inhibitors, Roche Diagnostics (Monza, Italy); serine/threonine phosphatase inhibitor and tyrosine phosphatase inhibitor, Sigma] and equal amounts of proteins were loaded, as determined by BCA Protein Assay kit (Thermo Scientific). SDS-PAGE and western blotting were performed following standard procedures. After incubation with primary antibodies, membranes were incubated with HRP-conjugated secondary antibodies and ECL Prime Western Blotting System (GE Healthcare) and subsequently imaged using a ChemiDoc imaging system (Biorad, Hercules, CA, USA). Densitometric analysis was performed with Image Lab software (Biorad). The following primary antibodies were used for western blotting: rabbit polyclonal anti-REST 1:1,000 (#07-579, Merck-Millipore, Darmstadt, Germany), rabbit polyclonal anti-calnexin 1:50,000 (#ADI-SPA-860, Enzo Life Sciences, Farmingdale, New York, USA), rabbit polyclonal anti-GFP 1:1,000 (#a11122, Invitrogen); mouse monoclonal anti-His 1:1,000 (#sc-57598, Santa Cruz Biotechnologies, Dallas, TX, USA).

### Immunocytochemistry, Immunohistochemistry and Confocal Imaging

Cells were fixed in PBS/4% paraformaldehyde (PFA) for 15 min and washed in PBS. Cells were permeabilized with 0.2% Triton-X 100 in PBS for 10 min at room temperature (RT) then incubated with primary antibodies diluted in PBS 1% BSA overnight at 4°C or 2 h at RT. After washes in PBS, cells were incubated with fluorescent secondary antibodies diluted in PBS 1% BSA. After washes, coverslips were mounted with Mowiol. For brain slices, mouse brains were dissected and fixed overnight in PBS/4% PFA. They were then cryoprotected in 20% and then 30% sucrose, embedded in OCT, frozen in isopentane (−55°C), and stored at −80°C. Coronal sections (18 μm) were cut with a cryostat and stored at −20°C before immunostaining. Sections were rehydrated in PBS for 5 min and then incubated in 1% Triton X-100 in PBS for 5 min. Slices were blocked for 1 h in PBS containing 5% BSA and incubated with primary antibodies for 24 h at 4°C. After washes in PBS, the slices were incubated with secondary antibodies for 2 h at RT, thoroughly washed and mounted on glass slides with Mowiol. All antibodies were diluted in PBS/5% BSA. Confocal images were obtained using a Leica SP8 confocal scan with a 40×/1.3 oil immersion objective and analyzed with the Leica LAS AF software (Leica Microsystem GmbH, Wetzlar, Germany).

The following primary antibodies were used for immunocytochemistry on fixed cells: rabbit polyclonal anti-GFP 1:500 (#a11122, Invitrogen); mouse monoclonal anti-His 1:200 (#ab18184, Abcam, Cambridge, UK). The following primary antibodies were used for immunohistochemistry on brain slices: rabbit polyclonal anti-REST 1:100 (#07-579, Merck-Millipore), mouse monoclonal anti-glial fibrillary acidic protein 1:1,000 (GFAP, #G3893, Sigma-Aldrich), mouse monoclonal anti-Neuronal Nuclei (NeuN, MAB377 Merck-Millipore). Fluorescently conjugated secondary antibodies were from Molecular Probes (Thermo-Fisher Scientific; Alexa Fluor 488, #A11029; Alexa Fluor 546, #A11030; Alexa Fluor 647, #A21450). Hoechst (#B2261, Sigma-Aldrich) was used to stain nuclei.

### RNA Extraction, Nanostring Analysis, and Real-Time PCR

Total RNA was extracted with the RNeasy^®^ Microarray Tissue Kit (Qiagen, Hilden, Germany) from the hippocampi of wild type mice expressing either the “closed” or the “open” probes. The corresponding cDNAs were prepared by reverse transcription of 1 μg of RNA using the SuperScript IV Reverse Transcriptase (ThermoFisher) with an oligo-dT primer according to the manufacturer’s instructions. The resulting cDNAs were used as a template for RT-qPCR using a C1000 Touch™ Thermal Cycler (BioRad) on a CFX96™Real-Time System following the manufacturer’s protocol. Relative gene expression was determined using the ΔΔCT method. To normalize expression data, primers for 10 commonly used housekeeping genes were used, and the normalization factor was determined using the geNorm software, as described (Vandesompele et al., 2002). This led to the selection of the following internal control genes in our assays: glyceraldehyde 3-phosphate dehydrogenase (Gapdh) and actin. Sequences of the primers used are listed in Supplementary Table S1.

For the Nanostring analysis of neuronal genes, fluorescently labeled probes were designed and synthesized by Nanostring Technologies (Supplementary Table S2). One hundred nanograms total RNA per sample, prepared as described above, was processed by Synlab Italia S.r.l. (Monza, IT) following standard procedures. Data were analyzed by using the nSolver Analysis Software Version 2.5.

### KA Injection and Seizure Scoring

To determine the dose-response of KA (Figure 2C), C57BL6/J mice were repeatedly injected with unitary doses of KA (5 mg/kg IP, in 0.9% saline) every 10 min and continuously monitored after each injection. Seizure scoring was conducted as described below. To induce seizures in transduced animals, mice received a single IP injection of vehicle or KA (25 mg/kg). In the KA-treated groups, behavioral seizures were evaluated off-line from video recordings taken during 1 h following the injection. Seizure scoring was conducted based on a modified version of Racine scale (Racine, 1972; McLeod et al., 2013) and the following parameters were considered: 0 = immobility, 1 = erratic twitches, 2 = straight tail, 3 = forepaws shaking, 4 = straight tail together with forepaw shaking (one time), 5 = continuously (>2) show an extended tail shake with forepaw shaking, 6 = display full tonic-clonic seizures. In case of severe attacks (severity score 6) the experiment and video recordings were stopped immediately after the attacks. For immunohistochemical and western blotting evaluation of REST induction, mice were sacrificed at the indicated times after KA administration. Dissection for mRNA extraction and Nanostring/qRT-PCR experiments was performed under a stereomicroscope equipped with a fluorescence lamp to isolate the GFP-expressing hippocampal tissue.

**Figure 1:**
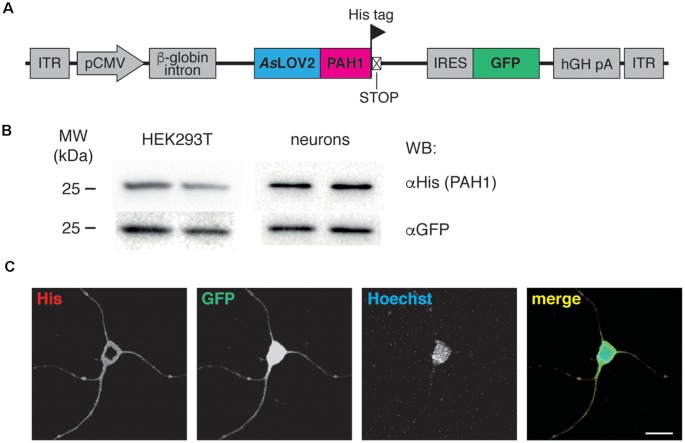
**(A)** Scheme of the AAV construct used throughout the study. ITR, inverted terminal repeats; pCMV, cytomegalovirus promoter; AsLOV2-PAH1, *Avena sativa* light-oxygen-voltage (LOV) domain 2 fused to the paired-amphipathic helix (PAH) domain 1 of the Sin3A protein; IRES, internal ribosomal entry site; GFP, green fluorescent protein; hGH pA, human growth hormone polyA. The positions of His tag and stop codon are indicated. **(B)** HEK293T cells were transfected with the AsLOV2-PAH1 construct, lysed and processed for western blotting using anti-His and anti-GFP antibodies, as indicated. Cortical neurons were infected at 7 DIV with recombinant AAV1/2 particles expressing AsLOV2-PAH1, lysed at 17 DIV and processed as described above. Duplicate lanes refer to two different cultures/experiments. **(C)** Cortical neurons were infected at 7 DIV with recombinant AAV1/2 particles expressing AsLOV2-PAH1, fixed at 17 DIV and processed for immunocytochemistry using anti-His (red channel), anti-GFP (green channel) and Hoechst to visualize nuclei (blue channel). Scale bar, 20 μm.

**Figure 2:**
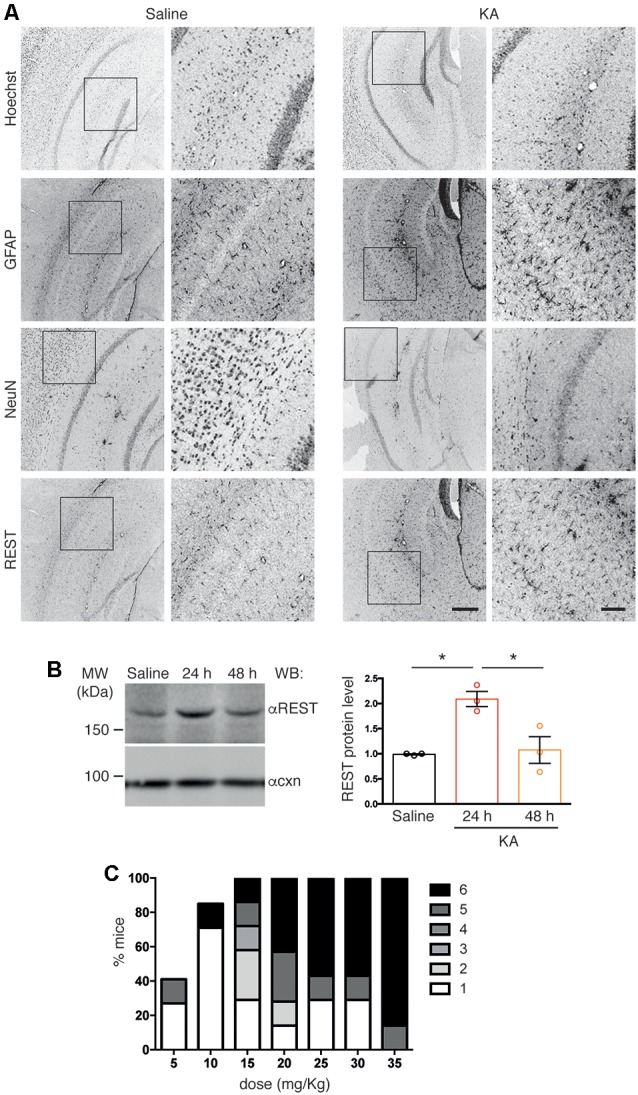
**(A)** Confocal images of coronal cortico-hippocampal slices derived from mice treated with either saline or kainic acid (KA; 25 mg/kg). Slices were labeled with anti RE-1 Silencing Transcription factor (anti-REST), anti-Neuronal Nuclei (anti-NeuN), and anti-GFAP antibodies, and with Hoechst to visualize nuclei, as indicated. High-magnification images corresponding to the boxed regions are shown for each staining. Scale bars: 300 μm in low-magnification images, 100 μm in high-magnification images. **(B)** The cortico-hippocampal region from saline- (control) or KA-injected mice was dissected at the indicated times after KA injection, lysed and processed for western blotting using anti-REST antibodies (left). Anti-calnexin antibodies were used to verify equal loading. Quantification of immunoreactive bands showed increased REST immunoreactivity at 24 h. One-way ANOVA (*F*_(2,6)_ = 12.06; *p* = 0.0079) followed by Tukey’s multiple comparison test (*p < 0.05); *n* = 3. **(C)** Increasing doses of injected KA (5 mg/Kg) in C57BL6/J mice induced seizures of increasing severity in a progressively higher percentage of animals. At 25 mg/Kg all animals reproducibly developed seizures.

### Statistics

Data are presented as means ± SEM throughout. D’Agostino’s and Pearson’s test were used to check the normality of the experimental data. The two-tailed unpaired Student’s *t*-test was used to compare two normally distributed sample groups, while one-way ANOVA followed by Tukey’s multiple comparison test was used to compare more than two normally distributed sample groups. For datasets of non-normal distribution, the Mann–Whitney *U*-test was used. The occurrence of a given behavioral trait in the mouse population was evaluated using the Chi-squared test. Alpha levels for all tests were 0.05% (95% confidence intervals). Statistical analysis was performed using GraphPad Prism 6 software (GraphPad Software Inc., San Diego, CA, USA).

## Results

### Cloning and Expression of AsLOV2-Based REST-Inhibiting and Control Probes

To study the impact of REST modulation on epileptogenesis, we developed a tool for its specific inhibition *in vivo*. The tool is composed of the PAH1 domain, fused to the photosensitive light-oxygen-voltage sensing (LOV) 2 domain of *Avena sativa* phototropin 1 (AsLOV2; Paonessa et al., 2016). PAH1 is the REST-interacting region of the mSin3 protein, which is part of the repressive REST complex (Grimes et al., 2000). We previously demonstrated that PAH1 is a competitive REST inhibitor (Paonessa et al., 2016). For *in vivo* transduction, we cloned the sequence coding for the His-tagged fusion protein into an AAV1/2 vector. In the construct, the expression of the probe is driven by the CMV promoter, while the expression of a GFP cassette is controlled by the IRES sequence, allowing the fluorescence detection of transduced neurons (Figure 1A). We verified the efficiency of transduction and the expression of the probe by immunoblotting of transfected HEK293T cells and transduced primary neurons (Figure 1B). The probe localized in the cytoplasm of neurons, as detected by immunocytochemistry and confocal imaging (Figure 1C).

### Kainic Acid Injection Induces a Transient Increase in REST Expression

Increased REST levels have been reported in several experimental models of epilepsy (Spencer et al., 2006; Hu et al., 2011). Here, we examined REST protein expression in the hippocampus upon pharmacological induction of epileptogenesis *via* IP injection of (KA, 25 mg/Kg), or saline as a control. We assessed REST protein levels by immunohistochemistry on coronal brain slices followed by confocal microscopy analysis. Slices were co-stained with anti-REST antibodies, anti-NeuN antibodies to detect neurons, anti-GFAP antibodies to detect astrogliosis associated with KA-induced epileptogenesis, and Hoechst to visualize nuclei. We detected increased REST immunoreactivity in slices from KA-treated animals compared to saline-treated control samples (Figure 2A, compare right and left panel). The specificity of the anti-REST antibodies was confirmed by the absence of signal in slices incubated with secondary antibodies only, omitting primary antibodies (not shown). The increase in REST expression peaked 24 h after KA injection, as revealed by immunoblotting of lysates obtained from dissected hippocampi (Figure 2B). At the dose used, KA induced seizures in all animals, in the majority of cases of severity 5/6 (Figure 2C).

### Chronic REST Inhibition Leads to Increased Neuronal Gene Expression

**Figure 3:**
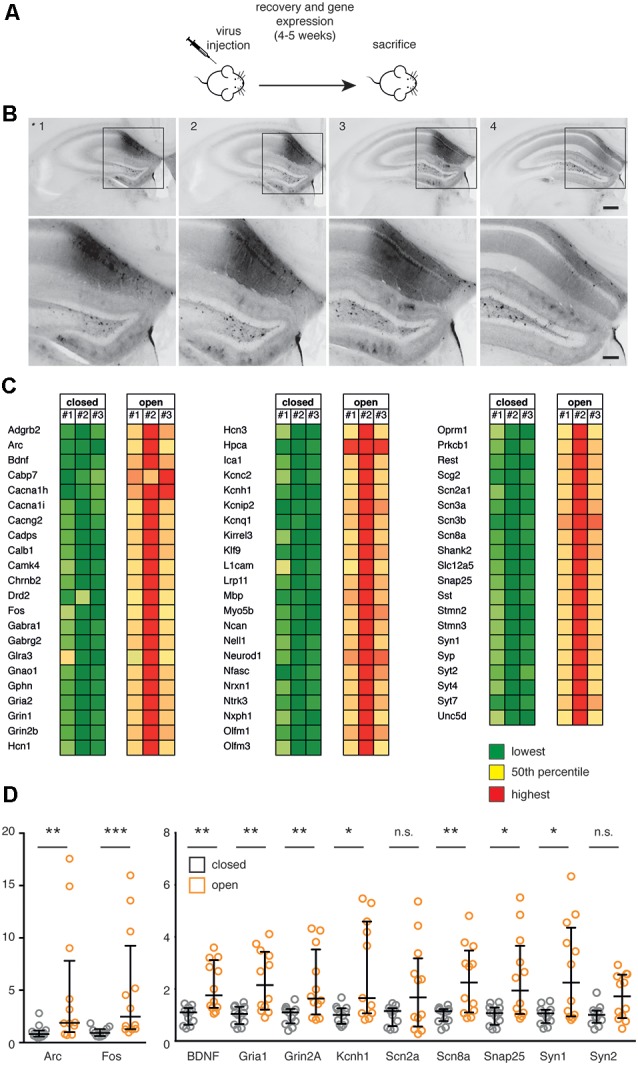
**(A)** Scheme of the *in vivo* experiment. C57Bl6/J male mice were subjected to stereotaxic injection within the hippocampus with recombinant AAV1/2 particles (0.65 μl-0.75 μl/hemisphere) expressing the constitutively closed [AsLOV2(C450A)-PAH1] or constitutively open [AsLOV2(I539E)-PAH1] probes, let recover for 4–5 weeks and sacrificed for gene expression studies. **(B)** Brain coronal slices (100 μm thickness) were prepared from paraformaldehyde (PFA)-fixed brain of AAV1/2-AsLOV2 injected mice (0.65 μl-0.75 μl/hemisphere), and transduced neurons in the hippocampus were detected by epifluorescence microscopy. Acquired images of four consecutive slices showed a high level of expression at the injection site, and a wide antero/posterior spread of the virus. Image 2 was taken at the injection site (from Bregma: AP 2.2, LAT 1.5; from brain: Z 1.65), the other images were acquired from slices at 100 μm intervals. Scale bars: 200 μm in low-magnification images, 100 μm in high-magnification images. **(C)** mRNA expression from the hippocampus of animals expressing either the constitutively open or the constitutively closed probe was analyzed using the Nanostring nCounter technology. We analyzed *n* = 3 animals per experimental group; a total of 64 REST-target (RE1-containing) genes were analyzed and the values were normalized against five housekeeping genes and for GFP expression. Results are color-coded separately within each gene: green color for low expression, red color for high expression. The complete list of genes and accession numbers is available in Supplementary Table S2;the expression values are available in Supplementary Table S3. **(D)** The expression of selected genes in the two experimental groups was validated by real time (RT)-PCR. Mann-Whitney test; n.s., non statistically significant (*p* > 0.05), *p < 0.05, **p < 0.01, ***p < 0.001; *n* = 12. Bars represent medians; whiskers represent interquartile ranges. The list of genes and accession numbers for RT-PCR is available in Supplementary Table S1.

To assess the functionality of our construct *in vivo*, AAV1/2-AsLOV2 recombinant viral particles (1 μl/hemisphere) were injected in the hippocampus of adult C57Bl6/J mice (Figure 3A) and epifluorescence images obtained from PFA-fixed coronal slices (100 μm) 1 month later. Efficient transduction of hippocampal neurons was detected with a wide (about 1.4 mm) anteroposterior diffusion of the virus from the injection site (Figure 3B). Furthermore, animals recovered quickly with no sign of damage at the injection site and no gross behavioral abnormalities, suggesting that the expression of the heterologous protein was well tolerated.

To assess the impact of REST inhibitory modulation by PAH1 on gene transcription, we employed the C450A (Salomon et al., 2000) and I539E (Harper et al., 2004) light-independent AsLOV2 variants to mimic the closed (inactive) and open (active) state of the AsLOV2 protein, in which the inhibitory peptide is hidden or exposed, respectively. One month after the AAV injection, hippocampi of transduced mice were dissected and a gene expression analysis was performed through the Nanostring nCounter technology (Geiss et al., 2008), which allowed us to quantitatively assess the expression of a panel of neuronal REST-target and non target genes (Supplementary Table S2). Interestingly, this analysis revealed a broad upregulation of all genes analyzed in animals expressing the active probe, compared to animals expressing the inactive probe (Figure 3C, Supplementary Table S3). Of particular interest, we observed the upregulation of synaptic genes (Gphn, L1cam, Nrx1, Shank, Snap25, Syn 1, Syp and several Syt), genes associated with inhibitory (Gabra1, Gabrg2, Glra3, Sst) and excitatory (Gria2, Grin1, Grin2b) transmission, and potassium channels (Hcn1, Hcn3, Kcnc2, Kcnh1, Kcnip2, Kcnq1). The changes in the expression of a subset of these genes and of some related genes were also analyzed through quantitative real-time PCR analysis, confirming the Nanostring results (Figure 3D). Altogether, these results suggest that mice expressing the open probe are characterized by an increased expression of a cluster of genes playing key roles in intrinsic excitability and synaptic transmission resulting from an inhibitory influence on the transcriptional repressor activity of REST.

### Chronic REST Inhibition Reduces the Susceptibility to KA-Induced Seizures

**Figure 4:**
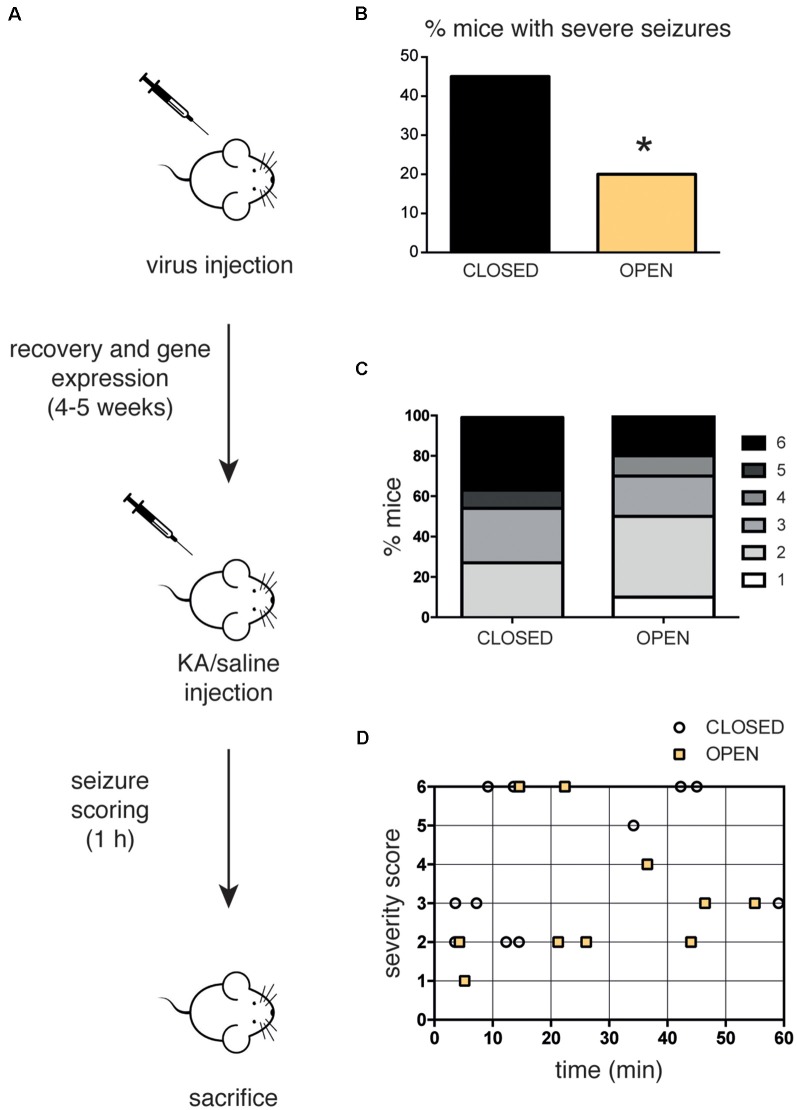
**(A)** Scheme of the *in vivo* experiment. C57Bl6/J mice (12–24 weeks old) were subjected to stereotaxic injection within the hippocampus of recombinant AAV1/2 particles (0.65 μl-0.75 μl/hemisphere) expressing either the constitutively closed [AsLOV2(C450A)-PAH1] or the constitutively open [AsLOV2(I539E)-PAH1] probes, let recover for 4–5 weeks and subsequently injected with kainate (KA, 25 mg/Kg) to induce seizures. Mice were continuously video-monitored for 1 h after KA injection to track and score seizures. Seizure severity was graded as described in the “Materials and Methods” section. **(B)** When compared to mice injected with the inactive variant, a low percentage of mice injected with the active variant showed severe seizure attacks (score 5/6). **(C)** Bar graphs indicate the percentage of animals for each severity score. Mice expressing the active probe displayed a mild phenotype when compared to control animals. **(D)** Single points represent the individual latency to the highest severity score. The majority of mice expressing the active probe experienced behavioral signs of seizures later than the animals expressing the inactive probe. *n* = 11 mice expressing the closed probe; *n* = 10 mice expressing the open probe. *p < 0.05; Chi-squared test.

To address the impact of REST inhibition on epileptogenesis, mice were injected with either the open or the closed probe and after 1 month of recovery, they received a single challenge dose of KA to induce seizures. After KA injection, mice were video-monitored for 1 h to detect seizure onset and severity, and quantified using a modified Racine scale (Figure 4A). Remarkably, animals expressing the active probe were less prone to the convulsant action of KA, showing a lower percentage of animals with severe attacks (classified as severity 5 or 6) and an overall reduced seizure score, compared to mice expressing the inactive control probe (Figures 4B,C). Interestingly, the analysis of the seizure latency suggests that animals with the active probe experience behavioral signs of the seizure later than mice expressing the inactive probe (Figure 4D). In particular, the majority of control animals developed behavioral alterations in the first 20 min, while the majority of mice with the open probe did it in the last 40 min of observation.

## Discussion

In this work, we have assessed the impact of long-term, mild inhibition of REST activity on the susceptibility to KA-induced seizures. To do that, rather than directly interfering with the REST-DNA binding, we adopted the strategy of preventing REST activation by mSin3 binding to its N-terminal domain. We previously described that a chimera of the minimal inhibitory peptide PAH1 with the switchable AsLOV domain was able to inhibit REST activity and de-repress the transcription of REST target genes in primary neurons when AsLOV was in the open state (unfolded Jα-helix) and the PAH1 peptide was accordingly exposed (Paonessa et al., 2016). To make this photoswitchable inhibition constitutively active, we generated a point mutant of AsLOV (AsLOV2^C450A^) in which the Jα-helix is permanently unfolded and used an alternative point mutant (AsLOV2^I539E^), in which the Jα-helix is permanently folded as a negative control (Paonessa et al., 2016).

As REST expression is increased in KA-induced seizures, we asked whether this increase has a compensatory or rather causal role in the epileptogenic activity triggered by KA. To this aim, the AsLOV2-PAH1 tool, which had been previously shown to work effectively *in vitro* (Paonessa et al., 2016), has been for the first time employed *in vivo*. AsLOV2-PAH1 was efficiently expressed in the hippocampus upon intracranial AAV injection and very well tolerated by the transduced mice, which did not show any gross behavioral alteration upon probe expression. The active probe was correctly working *in vivo* since the hippocampi of transduced mice expressing the active probe were characterized by moderate upregulation of all the REST-target genes, as expected by the chronic effective inhibition of REST repressor activity. Of note, we observed the upregulation of both REST-target and non-target genes, likely reflecting a cascade of effects whereby the initial de-repression of REST-target genes leads to a wider upregulation of neuronal genes. Among up-regulated genes there were clusters playing key roles in intrinsic excitability and synaptic transmission. Genes bi-directionally controlling intrinsic excitability included sodium (Scn2a1, Scn3a, Scn3b, Scn8a, Hcn1, Hcn3), calcium (Cacna1h, Cacna1i, Cacng2) and potassium (Kcnc2, Kcnh1, Kcnip2, Kcnq1) channels. Moreover, a large array of genes playing key roles in both inhibitory and excitatory synaptic transmission were activated, including (i) presynaptic actors of synaptic vesicle trafficking and exocytosis (Syn1, Syp, Syt2, Syt4, Syt7, Scg2, Sst); (ii) glutamate (Gria2, Grin1, Grin2, Shank2) and GABA/glycine (Gabra1, Gabrg2, Gphn, Glra3) receptor complexes; (iii) synaptic adhesion molecules (L1cam, Nrxn1, Nfasc); (iv) immediate early genes (fos, arc); (v) protein kinases involved in transcriptional control and homeostatic plasticity (Camk4; Map2k2, Map3k5, Prkcb1); (vi) neurotrophin signaling (Bdnf, Ntrk3); and (vii) calcium-binding molecules (Calb1, Hpca).

When mice expressing the open and close constructs were challenged with a single KA injection, animals expressing the active probe showed decrease propensity to develop seizures, which were also less severe. In fact, a lower percentage of animals expressing the active probe experienced tonic-clonic attacks, while the vast majority of them showed a mild phenotype, mainly characterized by tail extension and/or forepaws shaking. In addition, control mice displayed behavioral signs of seizure in a much shorter time, again indicating that mice transduced with the active probe were more resistant to the convulsive insult. Since the inhibition of REST activity was constitutive, the administration of the convulsant to trigger seizures found mice in a “low-REST”/“enhanced neuronal phenotype” state, i.e., with upregulation of many gene clusters. Such a global change in the transcriptional profile of neuron-specific genes controlling neuronal communication and network activity, as revealed by our gene expression analysis, is compatible with tighter control of intrinsic excitability and strengthening of both excitatory and inhibitory synaptic connections. This can render neuronal network less susceptible to external stimuli trying to shift the excitation/inhibition balance, by potentiating the push-pull control on depolarizing-hyperpolarizing conductances and on the balance between excitatory and inhibitory synaptic transmission and short-term plasticity. Moreover, the observed upregulation of K^+^ channels and of GABA/glycine receptors may represent a brake towards hyperexcitability, making neurons more refractory to the actions of convulsant drugs such as KA. Alterations of REST expression and/or activity have been associated with the onset of epilepsy, although the precise role of this factor in the progression of the pathology is still debated, and likely depends on the model employed and on the cell type analyzed (Hu et al., 2011; Liu et al., 2012; Patterson et al., 2017). Our findings are consistent with previous studies, which implicated REST in maintaining neuronal homeostasis and reducing the hyperexcitation of the network (Pozzi et al., 2013; Pecoraro-Bisogni et al., 2018; Zullo et al., 2019).

Our results support the notion that REST is actively contributing to the epileptic phenotype, as the chronic inhibition of its activity makes animals less prone to develop seizures upon KA challenge. Future studies will address with more specificity the cell type(s) where REST action is more prominent, by selectively expressing the probe in specific neural cell populations. Moreover, it will be crucial to identify more precisely the time window of REST inhibition that is sufficient to inhibit seizure development, so that appropriate strategies could be designed to exploit REST as molecular target for the treatment of paroxysmal neuropathologies characterized by network hyperexcitability.

## Data Availability Statement

All datasets generated for this study are included in the article/Supplementary Material.

## Ethics Statement

The animal study was reviewed and approved by the Italian Ministry of Health (Authorization # 73-2014-PR on Dec 5, 2014), and was carried out in accordance with the guidelines established by the European Community Council (Directive 2010/63/EU of 22 September 2010).

## Author Contributions

EC performed molecular cloning, AAV production, cell and primary neuron infection, biochemistry and confocal imaging, and RT-PCR analysis. FBu performed REST immunohistochemistry and immunoblotting on wild type animals. AR performed immunoblotting, supervised cell and molecular biology and biochemistry experiments. CM performed the *in vivo* experiments and seizure scoring. FC performed Nanostring analysis and wrote the article. FBe supervised the project and wrote the article.

## Conflict of Interest

The authors declare that the research was conducted in the absence of any commercial or financial relationships that could be construed as a potential conflict of interest.
