# Supplementary material for: Mild Inactivation of RE-1 Silencing Transcription Factor (REST) Reduces Susceptibility to Kainic Acid-Induced Seizures
Source: Front Cell Neurosci. 2020 Jan 10;13:580. doi: 10.3389/fncel.2019.00580 (PMC6965066; doi:10.3389/fncel.2019.00580)
Supplement: Supplementary file 4 [file Data_Sheet_1.docx]

Supplementary Material

**Supplementary Table 1.** List of primers used for real-time PCR. The table reports the following for each gene: (i) the gene name; (ii) the accession number; the sequence of the forward (iii) and reverse (iv) primers, (v) the full gene name. Data were further normalized for probe expression and visualized as box-plots (Fig. 3D).

**Supplementary Table 2.** NanoString nCounter code set design. Optimal sequences were designed on the chosen genes by Nanostring Technologies. The table reports the following for each gene: (i) the gene name; (ii) the accession number; the position (iii) and the sequence (iv) of the targeted region; (v) whether it is a housekeeping gene (HK); and (vi) the full gene name.

**Supplementary Table 3.** NanoString nCounter gene expression data. The table reports the following for each gene: (i) the gene name; (ii) the accession number; and (iv) the fold change of expression averaged per single animal. Values were processed by using the nSolver Analysis Software Version 2.5 (nanoString Technologies) by first removing the experimental background and subsequently normalizing to the HK genes. Data were further normalized for probe expression and visualized as a heat map (Fig. 3C).
